# Aortic Valve Stenosis, a Precipitating Factor of Recurrent Bleed in Colonic Angiodysplasia: A Literature Review

**DOI:** 10.7759/cureus.15903

**Published:** 2021-06-24

**Authors:** Adejoke M Johnson, Praise E Chovwen, Ezekiel J Akpan, Anna Patel

**Affiliations:** 1 Medicine, All Saints University School of Medicine, Roseau, DMA; 2 Medicine, All Saints University College of Medicine, Kingstown, VCT; 3 Gastroenterology and Hepatology, Community First Medical Center, Chicago, USA

**Keywords:** colonic angiodysplasia, arteriovenous malformations, heyde syndrome, refractory gastrointestinal bleed, aortic valve stenosis, aquired von willbrand disease

## Abstract

Angiodysplasia (also known as angioectasia) is a lesion characterized by abnormal, dilated small blood vessels in the mucosa and submucosal layers of the GI tract. With the estimated low incidence of active GI bleeding from these lesions, angiodysplasia can be challenging to diagnose. The presence of aortic stenosis has increased the recognition rate of angiodysplasia, especially in the elderly. Despite the associations between aortic stenosis and angiodysplasia (Heyde's syndrome) revealed in several studies, the etiology of Heyde syndrome is still debatable, which has led to the proposition of several hypotheses that are reviewed in this article. This activity will help review the meaning of Heyde's syndrome, epidemiology, proposed pathophysiology, diagnosis, and management by surveying articles published between 1955 and 2021 on PubMed. We used search terms such as "colonic angiodysplasia," "arteriovenous malformation," "Heyde syndrome," "refractory gastrointestinal bleed," "aortic valve stenosis," and "acquired von Willebrand disease." Findings revealed an association between aortic stenosis and lower gastrointestinal (GI) bleed.

## Introduction and background

Angiodysplasia, a common cause of gastrointestinal (GI) bleeding in the elderly, is the malformation of blood vessels in the GI tract. It is predominant in people aged 60 years and above. Age-related degenerative changes in the colon and strain on the bowel are proposed theories on the development of angiodysplasia in the elderly [1]. These lesions are usually <10 mm and proximal to the hepatic flexure (ascending colon) 80% of the time [2]. When occult GI bleeding is seen in patients younger than 40 years old, the etiology is generally not related to Heyde's syndrome [3]. In 1958, Heyde was the first to describe the association between chronic gastrointestinal bleeding and calcific aortic stenosis, birthing a set of medical signs and symptoms termed "Heyde's syndrome"[4].

## Review

Epidemiology

Heyde syndrome is characterized by GI bleeding in the presence of aortic stenosis. This association is detected incidentally in elderly patients. The reported prevalence of aortic stenosis in patients with gastrointestinal angiodysplasia ranges from 0% to 41% [5]. A study was done to highlight this association. Between 1990 and 2000, 73 patients diagnosed with angiodysplasia were identified. The prevalence of aortic stenosis was calculated as 31.7%, significantly higher than 14.0% noted in the comparison group (Mitral stenosis) with a p-value <0.001. Significant aortic stenosis was 2.6 times more common, while severe aortic stenosis was 4.1 times more common in patients with angiodysplasia than in the general population [6].

Pathophysiology 

The exact mechanism for the development of angiodysplasia in the setting of aortic stenosis is yet to be understood. However, multiple studied processes are involved in the etiology of Heyde's syndrome ranging from chronic venous obstruction, age-related degenerative processes, genetics, ischemia, Inflammation, and the dysfunction of an essential multimeric plasma glycoprotein called Von-Willebrand's factor.

Angiodysplasia is a common abnormality of blood vessels in the GI tract and the second leading cause of lower gastrointestinal bleeding in patients older than 60 years, with the first being diverticulosis [7]. According to Boley et al., intermittent intestinal submucosal venous outflow obstruction is more predominant in the elderly population and is part of the aging process [8]. This intermittent venous obstruction is associated with a transient increase in colonic lumen pressure and size. This increase leads to multiple episodes of increasing wall tension with obstruction of submucosal venous outflow, especially in areas where vessels pierce the muscularis propria of the colon. This process leads to a gradual dilation of the submucosal veins, venules, and arterioles supplying them. Eventually, the capillary rings dilate, the precapillary sphincters lose their competency and small arteriovenous communication forms. The above accounts for the characteristic early-filling vein observed during mesenteric angiography [7]. In addition to the venous outflow obstruction commonly seen in the elderly, age-dependent tissue degeneration of the aortic valve and vessels in the colon has also contributed to the association between aortic stenosis and angiodysplasia [9].

To understand the pathophysiology of Heyde's syndrome and its connection to the Von Willebrand factor (VWF), the basic science of the VWF needs to be understood. VWF is a multimeric globular plasma glycoprotein. The synthesis of the VWF occurs in the endothelial cells and megakaryocytes then stored in Weibel-Palade bodies and the alpha granules of platelets [10,11]. These granules contain ultra-large and large multimers of VWF that act as the most hemostatic forms of VWF and are rapidly released into the circulation when local hemostasis is needed. [12]. In the early 1990s, Warkentin suggested that the association between aortic stenosis and GI bleeding is linked to the deficiency of the largest VWF multimers [13]. In aortic stenosis, an increase in the velocity of the blood flowing through the valve leads to increased shear stress on the blood components. This higher stress causes the coiled VWF to unravel early and become an elongated protein with an unfolded A2 domain, a phenomenon that should occur only at the injury site. When VWF becomes non-globular, it is no longer resistant to proteolysis by ADAMTS13. VWF becomes accessible for ADAMTS13 to cleave and reduces its multimer sizes, rendering it inactive; this leads to a deficiency in VWF, causing hemostatic dysfunction [14]. The inactive VWF leads to bleeding in the vessels of the gastrointestinal system because platelets become unable to bind to damaged blood vessels (arteriovenous malformations) [13].

A study revealed that aortic stenosis could also contribute to the increased incidence of lower GI bleeding due to reduced gastrointestinal perfusion; however, this study was an observational case-control study without any histological association [15]. When the human tissue is in a hypoxic state, new blood vessels are formed in the colon (neovascularization) due to the increased expression of vascular endothelial growth (VEGF), pro‐angiogenic factors. These new vessels are prone to bleeding. Another study was done in the late '90s to investigate the contribution of angiogenesis to the pathogenesis of human colonic angiodysplasia. Immunochemistry of specimens using affinity-purified rabbit polyclonal antibodies and a streptavidin-biotin peroxidase method was used in 18 patients with colonic angiodysplasia. The study was done to evaluate the expression of VEGF alongside its receptors (Flt-1, KDR) and basic fibroblast growth factor (BFGF). Strong immunoreactive reaction for VEGF was noted to be proportionately distributed in the endothelium of blood vessels of all sizes in 16 (89%) specimens of colonic angiodysplasia. At the same time, minimal immunoreactivity was found in the normal colon. Vascular staining for flt-1 observed in eight (44%) patients with colonic angiodysplasia was not identified in patients with a normal colon. Vascular immunoreactivity for BFGF was identified in seven (39%) specimens from patients with colonic angiodysplasia. This study helps to reveal the development of angiodysplasia as a result of hypoxia induced by aortic stenosis [16]. The increased expression of VEGF in angiodysplasia lesions helps us understand how the novel drug thalidomide, an anti-angiogenic drug, aids in managing angiodysplasia.

A chronic inflammatory reaction is another proposed theory that is said to be involved in the development of angiodysplasia in the presence of aortic stenosis. A study revealed that T lymphocytes and the expression of the Interleukin-2 receptor were predominant in a stenosed aortic valve [17]. This same inflammatory reaction is also seen in angiodysplasia of the colon and could serve as a contributing factor.

Clinical presentation

As stated above, only about 10% of patients with angiodysplasia will have active bleeding. When symptomatic, presentation varies from non-specific occult bleeding to overt bleeding with significant blood loss (less common). The bleeding is often recurrent and intermittent. Patients with occult bleeding usually go undetected till symptoms of anemia, including fatigue, shortness of breath, syncopal/presyncopal episodes, parlor, or craving of non-nutritive substances (pica), occur. The underlying aortic stenosis also contributes to fatigue and shortness of breath. Patients can also present with acute-on-chronic exacerbation of GI bleeding requiring hospitalizations and multiple blood transfusions.

The frequency of these various clinical manifestations was evaluated in 41 patients who had endoscopies done for multiple reasons. On endoscopy, angiodysplasia lesions were identified. The lesions were associated with overt bleeding in 11 patients (27%) and occult bleeding in 9 (22%). The remaining 21 patients (51%) had no history of occult or overt bleeding, and the angiodysplasia was considered an incidental finding [18]. The probability of future bleed from incidentally found lesions is uncertain, but the number of lesions and the presence of comorbid coagulopathy or platelet disorders are major determining factors. In addition, patients that have had bleeding lesions have a higher risk of rebleeding [19]. In aortic stenosis, large-sized VWF multimers are affected; hence generalized bleeding, i.e., not localized to the colon, is less common because small and intermediate-sized VWF multimers remain available for hemostasis [13,20]. However, severe recurrent epistaxis has been reported [21].

Diagnosis

The specific diagnostic modality for identifying angiodysplasia of the GI varies on an individual level. The primary diagnostic modalities used include the following.

Endoscopic imaging is currently the primary modality used for the diagnosis of angiodysplasia. On endoscopic imaging, i.e., colonoscopy, angiodysplasia has the characteristic appearance of small (5 to 10 mm), flat, cherry-red lesions with a fern-like pattern of arborizing, ectatic blood vessels radiating from a central vessel. In the absence of an active bleed, visualized lesions cannot be diagnosed as the source of the bleed, especially when multiple lesions co-occur through the GI tract. Colonoscopy is routinely done to utilize advantages like biopsy sampling, marking lesions for surgery, and temporary control of acute bleed. The sensitivity of endoscopy is estimated to exceed 80% [22]. However, endoscopy may not always identify lesions. Therefore, repeat examinations are considered, especially in cases of high clinical suspicion and severe clinical presentations [23]. Angiodysplasia can also be identified incidentally when endoscopic imaging is performed for various other indications.

Radiologic imaging, helical computed tomography angiography (CTA), or magnetic resonance angiography (MRA) are second-line diagnostic modalities. They are used for primary diagnosis in patients with signs of active overt GI bleeding and confirm the bleeding location identified on colonoscopy. CTA and MRA are rapid in diagnosing acute GI bleeding but have limited indications due to lack of therapeutic ability, contrast allergy, and radiation exposure. Over 70% and 100% were the sensitivity and specificity of CTA, respectively [24]. 

Standard angiography is occasionally used to manage acute GI bleeding as bowel prep is not required (unlike endoscopic imaging). It shows a characteristic early venous filling, Standard angiography aids in specific localization of a lesion and therapeutic procedures simultaneously. Standard angiography is reserved for patients in whom other modalities have failed to identify the source of bleeding [25].

Additionally, extensive workup should be done in elderly patients presenting with any combination of aortic valve stenosis, gastrointestinal bleeding, or evidence suggestive of acquired Von-Willebrand syndrome. The initial focus should be placed on ruling out life-threatening conditions. Laboratory evaluation includes complete blood count, comprehensive metabolic panel, coagulation panel, fecal occult blood testing. ECG and echocardiogram are performed if the patient has had no recent cardiovascular workup available. Definitive decisions on the diagnostic pathway will be made based on individual clinical state and doctors' inclination [26].

Treatment

Management of angiodysplasia encompasses therapy that deals with submucosal bleeding and the breakdown of the VWF. Administration of desmopressin has the primary goal of increasing the VWF, although this form of treatment is temporary [27]. Desmopressin stimulates the release of the VWF from endothelial cells, thus increasing the amount of effective VWF multimers in the blood [28].

Ultimately, the course of therapy for Heyde's syndrome (endoscopic therapy versus surgical aortic valve replacement, SAVR) depends on the patient's presentation. Currently, there are no protocols or approved guidelines that have been agreed on as the management for Heyde's syndrome [26].

In acute submucosal bleeding, therapy focuses on maintaining blood pressure by blood transfusion, IV fluid infusion, and endoscopic therapy to stop gastrointestinal bleeding. In patients who have episodes of refractory bleeding, thalidomide can be administered while paying close attention to any adverse effects [29]. Definitive treatment is surgical replacement of the stenosed valve [30]. Surgical replacement could either be done by a transcatheter aortic valve replacement (TAVR) or SAVR. The replacement of the stenosed valve prevents the destruction of the VWF multimers by shear force. Surgical replacement treats the acquired clotting disorder and anemia from recurrent submucosal bleeding [31]. The effects of valve replacement have been described in the case of a 78-year-old female with diagnoses of minimal coronary artery disease, aortic stenosis, and resection of the sigmoid colon due to diverticulosis. She had multiple episodes of lower intestinal bleeding and coffee-brown vomitus. These episodes were attributed to angiodysplastic lesions found in the stomach and ascending colon following endoscopic procedures. Symptomatic management, including multiple blood transfusions, iron infusion, and endoscopic laser coagulation, was done to maintain her hematocrit level. These efforts were able to keep her hematocrit levels only temporarily due to recurrence of bleeding. She was referred for replacement of the aortic valve, and blood tests showed hemoglobin of 8.7 g/100 ml, prothrombin time of 10.5 seconds (range, 10.0 to 13.5 seconds), partial thromboplastin time of 26 seconds (range, 20 to 33 seconds), and platelet count was 298,000 per cubic millimeter. A follow-up after nine months, the patient revealed no recurrence of bleeding. Conservative therapy was discontinued as her anemia resolved. Blood tests after aortic valve replacement showed hematocrit and hemoglobin were 39.4% and 12.5 g/100 ml, respectively [32].

In patients unfit for surgery, conservative therapy like iron supplements and regular blood transfusion might be required to maintain hematocrit levels.

Prognosis

Prognosis is excellent in patients undergoing aortic valve replacement, which is curative to the clotting disorder [33]. In retrospective and prospective studies, there has been noted cessation of gastrointestinal bleeding in 79% to 93% of patients that undergo aortic valve replacement [34,35]. In patients who had repeat episodes of bleeding, there was a noted reduction in the number of bleeding episodes per year [34]. Patients have a similar risk of mortality when comparing TAVR to SAVR [36]. Patients unable to undergo aortic valve replacement have a poorer prognosis due to recurrent gastrointestinal bleeding from angiodysplastic lesions with 95% of patients having recurrent episodes of bleeding [35].

## Conclusions

Aortic stenosis and angiodysplasia are diseases that have a high prevalence in elderly patients. These diseases also commonly occur together with aortic stenosis resulting in exacerbation of bleeding from angiodysplastic lesions. This collection of symptoms known as Heyde's syndrome should be explored when pondering the causes of recurrent bleeding. Advancements in medical technology have made diagnosing and treating Heyde's syndrome easier and less invasive.
